# A comparative analysis between sequential boost and integrated boost intensity-modulated radiation therapy with concurrent chemotherapy for locally-advanced head and neck cancer

**DOI:** 10.1186/s13014-016-0756-x

**Published:** 2017-01-13

**Authors:** Gregory Vlacich, Mark J. Stavas, Praveen Pendyala, Shaeu-Chiann Chen, Yu Shyr, Anthony J. Cmelak

**Affiliations:** 1 Department of Radiation Oncology, Vanderbilt University Medical Center, Nashville, TN USA; 2 School of Medicine, Vanderbilt University Medical Center, Nashville, TN USA; 3 Center for Quantitative Sciences, Vanderbilt University Medical Center, Nashville, TN USA; 4 Vanderbilt Ingram Cancer Center, Vanderbilt University Medical Center, Nashville, TN USA; 5 Current affiliation: Department of Radiation Oncology, Washington University School of Medicine, 4291 Parkview Place, Campus Box 63110, St. Louis, MO 63110 USA

**Keywords:** Squamous cell carcinoma of the head and neck, Radiotherapy, Intensity modulated, Sequential boost, Acute toxicity

## Abstract

**Background:**

Planning and delivery of IMRT for locally advanced head and neck cancer (LAHNC) can be performed using sequential boost or simultaneous integrated boost (SIB). Whether these techniques differ in treatment-related outcomes including survival and acute and late toxicities remain largely unexplored.

**Methods:**

We performed a single institutional retrospective matched cohort analysis on patients with LAHNC treated with definitive chemoradiotherapy to 69.3 Gy in 33 fractions. Treatment was delivered via sequential boost (*n* = 68) or SIB (*n* = 141). Contours, plan evaluation, and toxicity assessment were performed by a single experienced physician. Toxicities were graded weekly during treatment and at 3-month follow up intervals. Recurrence-free survival, disease-free survival, and overall survival were estimated via Kaplan-Meier statistical method.

**Results:**

At 4 years, the estimated overall survival was 69.3% in the sequential boost cohort and 76.8% in the SIB cohort (*p* = 0.13). Disease-free survival was 63 and 69% respectively (*p* = 0.27). There were no significant differences in local, regional or distant recurrence-free survival. There were no significant differences in weight loss (*p* = 0.291), gastrostomy tube placement (*p* = 0.494), or duration of gastrostomy tube dependence (*p* = 0.465). Rates of acute grade 3 or 4 dysphagia (82% vs 55%) and dermatitis (78% vs 58%) were significantly higher in the SIB group (*p* < 0.001 and *p* = 0.012 respectively). Moreover, a greater percentage of the SIB cohort did not receive the prescribed dose due to acute toxicity (7% versus 0, *p* = 0.028).

**Conclusions:**

There were no differences in disease related outcomes between the two treatment delivery approaches. A higher rate of grade 3 and 4 radiation dermatitis and dysphagia were observed in the SIB group, however this did not translate into differences in late toxicity. Additional investigation is necessary to further evaluate the acute toxicity differences.

**Electronic supplementary material:**

The online version of this article (doi:10.1186/s13014-016-0756-x) contains supplementary material, which is available to authorized users.

## Background

Radiation therapy for locally advanced squamous cell carcinoma of the head and neck (LAHNC) has changed considerably over the past two decades with the advent of intensity modulated radiation therapy (IMRT). While the toxicity profile for patients with LAHNC has improved, significant acute and late toxicities remain. In the pre-IMRT era, definitive treatment consisted of conventional radiation at a constant dose-per-fraction with successive narrowing of fields to account for normal tissue tolerances. With newer IMRT techniques, one can differentially dose gross disease, high-risk subclinical, and low-risk subclinical disease without changing the overall treatment volume. This planning technique gained popularity because of improved planning efficiency (only a single inverse treatment plan is required), and early studies suggesting improved dose distributions [1, 2]. As a result, this method of IMRT, often referred to as simultaneous integrated boost (SIB), became the predominant mode of delivering radiation for LAHNC.

Studies have shown that dose-per-fraction to gross disease as high as 2.2 Gy/fraction with concurrent chemotherapy [3] and 2.4 Gy/fraction with radiation alone [4] with integrated boost are safe and effective. Conversely, a dose-per-fraction as low as 1.6 Gy/fraction shows equivalent tumor control to the more conventional 2.0 Gy/fraction [5]. Despite this working range, there has been variability among integrated boost regimens with regard to toxicity and outcome profiles [6].

As inverse treatment planning techniques continue to improve, a renewed interest in sequential boost planning has developed. To date, few studies directly examine the differences between sequential boost and SIB IMRT in the treatment of LAHNC. Purely dosimetric studies on limited patient numbers or patient surrogates have varied regarding the advantage of one technique over the other. Older studies suggested that SIB improved normal tissue sparing and/or improved conformality [1, 7, 8], while recent studies demonstrate reduced dose to normal structures in the neck, and improvement in conformality with sequential boost IMRT [9, 10]. Studies comparing clinical outcomes and toxicities among patients treated with either technique are lacking. A recent randomized study from Thailand explored toxicities and outcomes in a small cohort of nasopharyngeal carcinoma patients and showed no difference in acute toxicity rates or early outcomes between the two IMRT techniques [11]. However, the absence of more common head and neck cancer subtypes in this study and the low overall rate of grade 3 or higher toxicity compared to historical controls may limit the applicability of these results.

At our institution, a transition from SIB to sequential boost IMRT for LAHNC was intentionally made for all patients. This was based on clinical observations that many SIB patients developed acute radiation toxicities requiring treatment breaks or premature treatment completion and sequential boost was attempted to mitigate these effects. In this study, we evaluate the toxicities, survival, and recurrence rates associated with sequential boost IMRT versus SIB in consecutive cohorts of patients using similar concurrent chemotherapy.

## Methods

### Design and patients

We performed a retrospective chart review of consecutive patients who received definitive concurrent chemoradiotherapy with either SIB or sequential boost IMRT for LAHNC between April 2003 and February 2012. Eligibility included biopsy-proven AJCC Stage III-IVB squamous cell carcinoma of the head and neck for which curative surgical resection was not indicated or recommended. Stage II patients were included if they received comprehensive nodal irradiation with concurrent chemotherapy. Primary tumor sites included nasopharynx, paranasal sinus, oropharynx, oral cavity, hypopharynx, and larynx. Patients with unknown primary were also eligible if pharyngeal and nodal radiation was delivered. Patients were at least 18 years old with an Eastern Cooperative Oncology Group (ECOG) performance status of 0–2. Exclusion criteria included prior oncologic resection, chemotherapy, or radiotherapy for their head and neck cancer, or concurrent active malignancy. Patients with stage IVC disease at diagnosis were excluded except those with regional metastases amenable to treatment with curative intent. This study was approved by the Institutional Review Board of Vanderbilt University.

Because there was an intentional and comprehensive shift from SIB to sequential boost by the treating radiation oncologist, the cohort of eligible patients treated with either technique are not overlapping and include all patients regardless of stage, subsite, or specific disease characteristics (e.g. bulky or extensive lymphadenopathy). Eligible patients were treated with SIB from approximately 2003 to 2008 and with sequential boost from approximately 2009 to 2012, thus accounting for the relatively larger SIB cohort and the variable follow up between groups.

### Radiation treatment planning and dose-volume analysis

Radiation was delivered using IMRT. Patients underwent CT-based simulation with immobilization using a custom thermoplastic mask (Aquaplast, Wycoff, NJ). Imaging studies and clinical exam with endoscopy were utilized in tumor delineation. Based on these evaluations, a gross tumor volume (GTV) was generated. Clinical target volume (CTV)/planning target volume (PTV) expansion of the primary GTV involved individualized expansions based on location and specific at-risk sites of subclinical disease, but generally involved a composite margin of 1.0–1.5 cm around the GTV. Lymph node levels were contoured based on the Radiation Therapy Oncology Group consensus guidelines and routinely included bilateral level II–IV lymph nodes [12]. For involved nodal levels, the entire level as defined above was treated to the gross tumor prescription dose. If involved at presentation, Level I and/or V lymph nodes were also included and were covered prophylactically as clinically indicated. For patients with unknown primary, comprehensive nodal and pharyngeal irradiation was performed. Treatment volume definitions and expansions were consistent between SIB and sequential boost groups.

Normal and avoidance structures were typically contoured based on their anatomic definitions. Skin was contoured as 3–5 mm from the surface. Treatment planning was then performed using Eclipse treatment planning software (Varian Medical Systems, Palo Alto, CA) and plans evaluated using dose-volume histograms. Dose to ≥ 95% of the target volume was required to be within ± 5% of the prescribed dose. Dose to ≥ 95% of the prophylactically treated nodal volume was required to be within + 8% to −5% of the prescribed dose. The maximum dose to the spinal cord and brain was required to be <4500 cGy and the median dose to total parotid gland tissue was required to be <2000 cGy. For non-laryngeal tumors, treatment plans attempted to achieve a dose ≤ 2000 cGy to 50% of the laryngeal volume and a maximal dose to the pharyngeal constrictors <5000 cGy to minimize toxicity. Dose and volume characteristics for organs at risk including the larynx, base of tongue and esophagus were documented for each individual and compared.

### Radiation dose and fractionation

For sequential boost radiation, the tumor and involved and prophylactic nodal volumes all received 50.4 Gy at 2.1 Gy/fraction. Gross tumor and involved nodal volumes then received an additional 18.9 Gy at 2.1 Gy/fraction for a total dose of 69.3 Gy in 33 fractions. For SIB, tumor and involved nodal volumes received 69.3 Gy in 2.1 Gy/fraction as above, and prophylactic nodal levels received 1.7 Gy/fraction to 56.1 Gy in 33 fractions. Treatment was delivered via a Varian Clinac 2100 or Varian Trilogy with 6 MV photons and 7 to 9 radiation fields using a step-and-shoot method with sliding window technique. VMAT was not utilized in either cohort. Treatment was given once a day for 5 consecutive days each week. Treatment plans were required to meet coverage thresholds and normal tissue constraints as described above. All IMRT treatment plans underwent independent departmental review.

### Chemotherapy

Induction chemotherapy was used at the discretion of the treating medical oncologist, and it was typically offered to patients with a bulky tumor, N3 disease, or prominent level IV or V lymph nodes. In both cohorts, induction chemotherapy consisted of weekly paclitaxel 60 mg/m^2^ and carboplatin area under the concentration-time curve (AUC) 2 for a total of 9 weeks for most patients. Other regimens were rarely utilized as described in Additional file 1: Table S1. All patients in the SIB cohort and the vast majority in the sequential boost cohort received concurrent weekly paclitaxel 30 mg/m^2^ and carboplatin AUC 1 given with approximately 7 weeks of radiation.

### Assessment of complications

Toxicities were graded according to the current version of the Common Terminology Criteria for Adverse Events at the time of treatment (http://ctep.cancer.gov/protocolDevelopment/electronic_applications/ctc.htm). Acute radiation toxicities were graded weekly in a prospective manner by a single treating radiation oncologist, and a numerical grade was recorded directly at the time of evaluation. “Grade 0” was also utilized prospectively in any category when there were no clinical findings or reported symptoms. When no numerical grade was recorded and insufficient data was available to assign a grade, no data was recorded for that week. The highest-grade toxicity achieved during the course of radiation was used for analysis. All patients were evaluable in the sequential boost cohort. All patients in the SIB cohort were evaluated for treatment outcome, but toxicity analysis was limited to patients treated at Vanderbilt University Medical Center (and not our satellite facilities) (*n* = 88). For each acute toxicity, there was insufficient data to reliably assign *any* grade during the entire course of treatment in a small fraction of patients from each cohort. Subsequent analyses were limited only to patients with available data, thus accounting for the variable *n* values in Table 3.

### Follow-up

Follow-up was performed as described previously [13]. Biopsy of suspicious lesions was performed to formally establish treatment failure. Consensus option of a multidisciplinary tumor board was required to establish treatment failure in the absence of biopsy.

### Statistical methods

Data were analyzed using R version 3.1.0. Overall survival (OS) was defined as the time from diagnosis to death from any cause. Disease-free survival (DFS) was defined as the time from diagnosis to any type of recurrence or death from any cause. Local or regional recurrence was defined as recurrence at the primary site or nodal sites respectively. OS, DFS, local and regional recurrence-free survival, and distant disease-free (or metastasis-free) survival were estimated using the Kaplan-Meier statistical method. Differences between sequential and integrated boost cohorts were evaluated using the Log-Rank test. Univariate analysis with Log-Rank tests was used to identify significant prognostic variables for DFS. Multivariate analysis was performed using the Cox proportional hazards model. For comparison of acute toxicity rates between cohorts, the Wilcoxon rank sum test for continuous/ordinal variables was utilized and the Pearson chi-square test was used to compare the incidence of “low” (grade 0–2) and “high” (grade 3 and 4) grade toxicity between cohorts. Dosimetric variables were compared using descriptive statistics and two tailed student t-test. *p* values <0.05 were considered statistically significant.

## Results

### Patient and tumor characteristics

Patient and tumor characteristics are listed in Table 1. Sixty-eight patients received concurrent chemoradiation with sequential boost IMRT and 141 patients received SIB between April 2003 and February 2012. The median age at diagnosis was 60 (range, 30 to 75) in the sequential cohort and 57 (range, 37–88) in the SIB cohort. Median follow up in the sequential boost cohort is 30.6 months (range, 6.1 to 54.7 months for surviving patients), whereas the SIB cohort, upon update from initial analysis [13], had a median follow up of 57.8 months (range, 2.4 to 97.1 months). There was a male predominance in both groups, and the majority of patients had stage IVA disease and primary oropharyngeal cancer. The sequential boost cohort had a larger percentage of oropharyngeal tumors (and less nasopharynx and oral cavity) and a higher proportion of patients with an ECOG performance status of 2 (13% vs. 3%). Otherwise, the cohorts were well matched.

**Table 1:** Patient and tumor characteristics

	Sequential boost cohort	Simultaneous integrated boost cohort
Characteristic	Number (%) *N* = 68	Number (%) *N* = 141
Sex		
Male	56 (82)	122 (87)
Female	12 (18)	19 (13)
Age (Median)	60 (30–75)	57 (37–88)
Initial primary tumor site		
Hypopharynx	2 (3)	6 (4)
Larynx	15 (23)	30 (21)
Nasopharynx	1 (1)	12 (9)
Oral Cavity	1 (1)	6 (4)
Oropharynx	48 (71)	81 (58)
Paranasal Sinus	0 (0)	2 (1)
Unknown primary	1 (1)	4 (3)
AJCC stage		
Stage II	2 (3)	3 (2)
Stage III	15 (23)	37 (26)
Stage IVA	44 (64)	83 (59)
Stage IVB	5 (7)	18 (13)
Stage IVC	2 (3)	0
ECOG Performance Status		
0	20 (30)	39 (28)
1	39 (57)	97 (69)
2	9 (13)	5 (3)
Smoking Status		
Current	25 (37)	65 (46)
Former	23 (34)	48 (34)
Never	20 (29)	28 (20)

### Feasibility

Induction chemotherapy was administered to 78% of patients in the sequential boost and 68% of patients in the SIB cohorts (*p* = 0.14). All patients received weekly chemotherapy concurrent with IMRT, with all (100%) in the SIB cohort and nearly all (95%) in the sequential boost cohort receiving weekly carboplatin/paclitaxel. Alternative induction and concurrent chemotherapy regimens are listed in Additional file 1: Table S1. Two sequential boost patients (3%) required a >5 consecutive day break from radiation treatment, similar to the 5% observed in the SIB cohort [13]. A greater percentage of patients in the sequential boost cohort received 4 or less cycles of concurrent chemotherapy (24% vs. 4%, *p* < 0.001). Reasons for not being able to complete 5 or more cycles were varied and included hematologic toxicity, patient compliance, and/or declining performance status in addition to delays from radiation-related toxicity.

### Dose and volume characteristics

Dose and volume characteristics for the larynx, base of tongue, and esophagus are reported in Table 2. The average volume of structure contoured, mean dose, max dose, and percent volume receiving 30 and 70 Gy were analyzed. No differences in contoured volumes or dose delivered to those volumes were considered to be statistically significant between the two cohorts.

**Table 2:** Comparative Dose-volume characteristics between sequential boost and integrated boost plans

Base of Tongue	Sequential boost	SIB	*p* value
Volume	13.2 cc	12.5 cc	*p = 0.13*
Mean	60.2 Gy	65.2 Gy	*p = 0.63*
Max	72.8 Gy	71.8 Gy	*P = 0.85*
V30	92.2%	97.5%	*p = 0.58*
V70	41.3%	47%	*p = 0.09*
Larynx			
Volume	25.79 cc	26.8 cc	*p = 0.08*
Mean	49.4 Gy	43.8 Gy	*p = 0.06*
Max	74.3 Gy	73.6 Gy	*p = 0.31*
V30	72%	60%	*p = 0.06*
V70	10.7%	19.5%	*p = 0.06*
Esophagus			
Volume	7.8 cc	6.9 cc	*p = 0.09*
Mean	25.6 Gy	27.2 Gy	*p = 0.19*
Max	54.0 Gy	49.2 Gy	*p = 0.08*
V30	34.6%	37.5%	*p = 0.31*
V70	0.3%	0%	*p = 0.08*

### Toxicity

There were no treatment-related deaths in either cohort. The percentage of patients requiring a modification to their radiation treatment (i.e. a break *or* an incomplete course) was comparable between the sequential boost and SIB cohorts (44% vs 43%, *p* = 0.91). However, a greater percentage of the SIB cohort did not receive the total prescribed dose due to reported acute toxicity (7% versus 0%, *p* = 0.028). There were no significant differences in the rate of gastrostomy tube placement (62% in sequential boost vs 67% in SIB) or in the rate of prolonged (>12 month) gastrostomy tube dependence (9% vs 12%).

Rates of common acute radiation-associated toxicities in the sequential and integrated boost cohorts are listed in Table 3. When comparing rates of “low” (grade 0–2) versus “high” (grade 3–4) grade acute toxicity, there were higher rates of grade 3/4 toxicity in the SIB cohort, with more grade 3/4 dermatitis (*p* = 0.012), dysphagia (*p* < 0.001) and a trend towards increased mucositis (*p* = 0.09). There were no differences in the rate of high-grade xerostomia. When limiting the analysis to individuals who completed ≥ 5 cycles of concurrent chemotherapy, the increased rate of high-grade dysphagia in the SIB cohort remained significant (*p* = 0.002), but the rate of high-grade dermatitis was comparable (*p* = 0.13). Interestingly, the increased rate of high-grade mucositis became statistically significant in the SIB cohort (*p* = 0.039) (see Additional file 1: Table S2).

**Table 3:** Analysis of acute toxicity in sequential and integrated boost cohorts

Sequential boost	Grade 0	Grade 1	Grade 2	Grade 3	Grade 4
Dermatitis (*n* = 64)	0%	2%	40%	56%	2%
Mucositis (*n* = 63)	3%	8%	19%	68%	2%
Xerostomia (*n* = 60)	12%	53%	33%	2%	0%
Dysphagia (*n* = 65)	3%	5%	37%	55%	0%
Weight loss (*n* = 64)	−14.25 lbs. (−1.6– −32.8)				
% Weight loss	8.1% (0.8–23.0)				
Simultaneous integrated boost	Grade 0	Grade 1	Grade 2	Grade 3	Grade 4
Dermatitis (*n* = 76)	0%	7%	15%	71%	7%
Mucositis (*n* = 73)	1%	3%	14%	79%	3%
Xerostomia (*n* = 70)	0%	56%	44%	0%	0%
Dysphagia (*n* = 75)	0%	1%	17%	79%	3%
Weight loss (*n* = 88)	−12.65 lbs. (+14.5– −33.0)				
% Weight loss	7.4% (0–18.3)				

### Treatment outcome

**Fig. 1:**
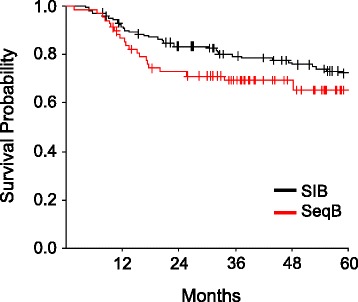
Kaplan-Meier estimates of overall survival for integrated boost (*black*) and sequential boost (*red*)

**Fig. 2:**
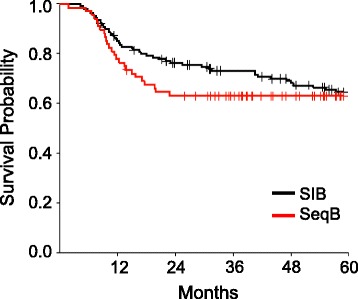
Kaplan-Meier estimates of disease-free survival for integrated boost (*black*) and sequential boost (*red*)

**Fig. 3:**
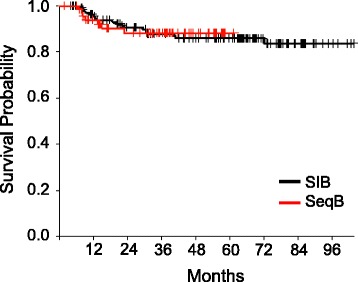
Kaplan-Meier estimates of local recurrence-free survival for integrated boost (*black*) and sequential boost (*red*)

**Fig. 4:**
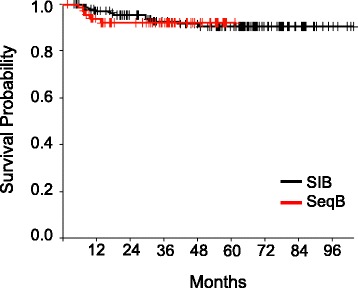
Kaplan-Meier estimates of regional recurrence-free survival for integrated boost (*black*) and sequential boost (*red*)

**Fig. 5:**
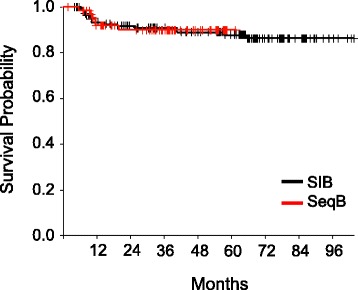
Kaplan-Meier estimates of metastasis-free survival for integrated boost (*black*) and sequential boost (*red*)

The overall outcomes of treatment are shown in Table 4. Median overall survival has not been reached for either cohort. At 4 years, overall survival (OS) was 69.3% in the sequential boost cohort and 76.8% in the SIB cohort (*p* = 0.13) (Fig. 1) and disease free survival (DFS) was 63 and 69% respectively (*p* = 0.27) (Fig. 2). Though not statistically significant, the decreased OS and DFS in the sequential boost cohort at the early time points may be due in part to the higher age and rates of death from intercurrent disease in this cohort (16% vs 12%). Regardless, the survival curves begin to converge by 48 months. There was no difference in the local-, regional-, or distant recurrence-free survival and the Kaplan-Meier curves are superimposed at essentially all time-points (Figs. 3, 4 and 5).

**Table 4:** Overall performance of patients treated with sequential boost and integrated boost at 4 years

4 year follow up	Sequential boost	Integrated boost
Overall survival	69.3% (56.5–79)	76.8% (68.6–83.1)
Disease Free Survival	63% (50.4–73.3)	69% (60.4–76.1)
Local recurrence-free survival	88.2% (76.7–94.2)	85.9% (78.2–91)
Regional recurrence-free survival	92.1% (82.1–96.7)	91.6% (84.8–95.4)
Distant disease-free survival	89.9% (78.8–95.4)	88.9% (82–93.3)

Though p16 status was only known for a small portion of patients in the sequential boost cohort, survival for the 9 patients that were confirmed to be p16-positive was approximately 89% at 4 years and, as expected, improved over the patients with negative p16 status or the unselected patient average.

## Discussion

The widespread use of IMRT for definitive treatment of LAHNC has resulted in an overall move from sequential boost to integrated boost radiation planning. This shift was influenced by early dosimetric studies suggesting improved dose distribution, initial ease of designing one plan versus two or more, and the predominance of using the SIB technique in Cooperative Group studies [14, 15]. At our institution, we made a conscious shift away from SIB to sequential boost IMRT for LAHNC based on our clinical observation that patients treated with SIB experienced more severe acute skin and pharyngeal toxicity and seemed less likely to complete treatment. Here, we systematically examined the outcomes and acute toxicities of comparable cohorts of consecutive patients who received definitive chemoIMRT for LAHNC either with SIB or sequential boost techniques. This is the first study comparing IMRT techniques among relatively large cohorts that include the more predominant head and neck subsites and were consecutively treated using similar chemotherapy and dose-volume constraints. Overall, we find that these techniques appear equivalent with respect to treatment outcomes though sequential boost IMRT is associated with some improvement in rates of acute toxicity.

Specifically, dysphagia was consistently improved in the sequential boost cohort. Dermatitis and mucositis rates were also improved, however statistical significance was variable depending on the group or sub-group analyzed. For instance, among the entire sequential boost cohort, rates of high-grade dermatitis were improved and high-grade mucositis were statistically comparable to SIB, while among only patients that completed most of their concurrent chemotherapy, this was reversed. The paradoxical effect with mucositis slightly improving (70% vs 68% grade 3/4) and dermatitis worsening (58% vs 66% grade 3/4) as more cycles of concurrent chemotherapy were completed in the sequential boost cohort is, in part, the consequence of chemotherapy being held for high-grade mucositis. Patients receiving less chemotherapy for this reason will of course have worse mucositis which prompted the initial decision to hold systemic therapy, but may also have improved dermatitis as a consequence. Additionally, the severity of dermatitis (more so than dysphagia and mucositis) can vary depending on factors independent of treatment volumes and disease, such as body habitus.

These differences in acute toxicity do not appear to be the results of variable dose and dose-volume parameters to key structures between SIB and sequential boost treatments. Instead, we postulate that the observed improvement in dermatitis and dysphagia seen clinically with sequential boost is due to decreasing radiation exposure to the lower neck and pharyngeal constrictors from 7 to 5 weeks. As studies have shown that 1.6 Gy/fraction results in equivalent tumor control with 2.0 Gy/fraction [5], early responding tissues with a similar α/β ratio such as mucosa and dermis should conversely have a comparable extent of damage from and recovery between fractions [16]. Therefore, doses within this range of 1.6–2.0 Gy should not significantly alter the overall effect on acute toxicity on a per-fraction basis. However, with fewer total fractions to normal tissue with sequential boost IMRT, the result would be decreased severity of acute toxicity.

The addition of concurrent chemotherapy to definitive radiation for locally advanced head and neck cancer is well known to increase toxicity compared to radiation alone [17–19]. Concurrent chemotherapy regimens are variable, but historically these have been cisplatin based or have included 5-FU. In our cohort, radiation was given with concurrent weekly carboplatin/paclitaxel and many also received weekly induction chemotherapy with the same agents. While cisplatin and carboplatin/paclitaxel have not been directly compared in a randomized fashion, this combination is often utilized as primary induction or concurrent chemotherapy or for patients unable to tolerate cisplatin, and our group and others have reported comparable treatment outcomes and acceptable toxicities [13, 20–22]. Furthermore, rates of high grade toxicity with concurrent carboplatin/paclitaxel seen in our SIB cohort are comparable with rates reported with cisplatin or 5-FU-containing regimens [13, 15, 17, 19]. Nevertheless, whether the differences seen in acute toxicity between SIB and sequential boost in our study would apply to patients receiving traditional cisplatin-based chemotherapy is not entirely clear and would require further evaluation.

A majority of patients in both cohorts also received induction chemotherapy prior to chemoradiation. Induction carboplatin/paclitaxel was typically offered to individuals with bulky tumors or lymphadenopathy and/or lower cervical lymphadenopathy, and was previously shown to be well-tolerated with 15% or less grade 3 and 0–1.5% grade 4 hematologic toxicity [13]. The potential impact of induction chemotherapy on subsequent toxicity during chemoradiation is difficult to ascertain in our study given the small fraction of patients who did not receive induction. However, the PARADIGM trial reported no difference in rates of mucositis, pain, xerostomia, or feeding tube dependency between those who received induction chemotherapy and those who did not [23]. Additionally, a recent meta-analysis confirmed that induction chemotherapy had no impact on rates of non-hematologic toxicity during the chemoradiation portion of therapy as compared to chemoradiation alone [24]. Furthermore, we found no significant difference in the utilization of induction chemotherapy between our SIB and sequential boost cohorts. This suggesting that induction chemotherapy is unlikely to contribute significantly to rates of acute toxicity reported in our study or the difference observed between cohorts.

As with chemotherapy regimens, there is often some degree of variability in radiation dose and fractionation used for definitive treatment of LAHNC. In our cohort, gross disease was treated to 69.3 Gy in 2.1 Gy per fraction. This is slightly hypofractionated when compared to the traditional 70 Gy in 2 Gy per fraction, however our dose and fractionation is similar to that used for concurrent treatment in nasopharyngeal carcinoma cooperative studies and results in comparable BED_3_ and BED_10_ to 70 Gy in 2 Gy fractions [15]. Additionally, dose to the prophylactic volumes is within the standard range for both the SIB and sequential boost groups. For these reasons, we feel that our reported results are applicable to other common LAHNC radiation doses and fractionations.

Most prior studies comparing SIB and sequential boost IMRT for LAHNC examined the differences from primarily a dosimetric standpoint. To our knowledge, our study is the largest to date exploring the *clinical* consequences of these distinct treatment approaches and the first to examine this difference among the most common head and neck subtypes. Recently, Songthong et al. reported on their phase II/III trial comparing SIB and sequential boost IMRT in patients with nasopharyngeal carcinoma and found no significant difference in short-term treatment outcomes or acute toxicities [11]. While the prospective nature of this study circumvents some of the limitations of ours and appears to contradict our findings with regard to toxicity, a number of factors call into question the strength of their conclusions and broader applicability. First, they utilized a different dose/fractionation scheme to gross disease in the SIB and sequential boost arm (2.12 Gy versus 2.0 Gy per fraction). Second, the study was limited to patients with nasopharyngeal carcinoma, which is significantly less common than oropharyngeal and laryngeal carcinoma. Third and most importantly, Songthong et al. report very low rates of grade ≥3 acute toxicity in either group when compared to historical controls (2–15% vs approximately 70%) [11, 15, 17, 19]. Furthermore, the study was powered to detect a generous 20% difference in acute toxicity rates yet did not reach the number of enrolled patients to achieve that (122 versus target of 218) [11]. With unusually low rates of high grade toxicity at less than 20% and effectively being powered to detect greater than a 20% difference, the lack of a significant difference may be due to the fact that this study was underpowered. In contrast, we report on a larger cohort with rates of grade ≥3 acute toxicity more in keeping with historical controls and were able to see a significant difference in various acute toxicities.

In general, SIB and sequential boost IMRT offer directly competing advantages and disadvantages with regard to radiobiology and treatment planning. Relative to SIB, sequential boost IMRT allows for consistent dose-per-fraction between all treatment volumes and throughout the course of treatment. This addresses the theoretical concern of under-dosing target volumes with 1.6–1.7 Gy fractions, particularly in the latter weeks of treatment when accelerated repopulation of tumor clonogens is prevalent [25, 26]. Additionally, sequential plans can better account for changes in body habitus (weight loss), edema, and tumor shrinkage when a new CT is obtained for boost planning. On the other hand, sequential boost IMRT is more time consuming and involves the summation of 2 or more treatment plans, which can theoretically result in more potential uncertainty in true dose distribution (particularly if based on separate CT simulations). Centers with high volume and/or limited or inexperienced staff find this more challenging compared to a single SIB plan. Continuous improvements in treatment algorithms and decreased time required for planning have improved the overall throughput for sequential boost IMRT, though these relative challenges still remain. Nevertheless, our data suggest that despite their differences, these two techniques are comparable with regard to treatment outcome.

As a retrospective analysis on two cohorts of patients treated at distinct periods of time, this study is limited by the lack of a prospective comparison of the two methods of treatment planning. These differences are partly mitigated by the use of consecutive patients, similar chemotherapy regimens, similar dose-volume constraints for IMRT, and prospective grading of toxicity by a single, experienced radiation oncologist at the time of treatment. The latter greatly minimizes the potential for intra- and inter-observer variability in these more subjective values. Furthermore, while the SIB and sequential boost patients were treated during non-overlapping intervals of time between 2003 and 2012 with the SIB group treated earlier, any differences in treatment planning, evaluation, and delivery were minimized over that span of time due to a number of factors. Specifically, contouring practices, treatment machines, dose calculation algorithms, and treatment planning software were consistent during this time. Still, there may be less quantifiable changes between these time periods that cannot be adequately accounted for. Additionally, changes in acute toxicity were not associated with gastrostomy placement or duration, weight loss, or changes in late toxicity in our cohort. Therefore, all these potential differences will need to be accounted for or confirmed in a formalized, prospective manner.

## Conclusions

Concurrent chemoradiation with sequential boost IMRT for LAHNC is well-tolerated and results in treatment outcomes comparable to simultaneous integrated boost. Our institutional experience also suggests that sequential boost reduces high-grade acute toxicity, and these data warrant further evaluation in a prospective study. In this setting of a potential reduction in acute toxicity, sequential boost IMRT is especially appealing as an alternative to SIB as image-guidance usage increases, particularly on-board cone beam CT. This could potentially reduce toxicity even further through real-time, clinically-guided modification of target volumes. Coupling image-guided assessment of tumor response with sequential field size reductions may allow for truly adaptive radiotherapy in a way not possible or practical with simultaneous integrated boost.

## Additional file

Additional file 1: Table S1. Chemotherapies Utilized in Sequential Boost Cohort. **Table S2.** Acute toxicity in sequential and integrated boost cohorts among patients who received 5 or more cycles of concurrent chemotherapy. (DOCX 12 kb)

## Abbreviations

5-FU: Fluorouracil
AJCC: American Joint Committee on Cancer
AUC: Area under the curve
CT: Computed tomography
CTV: Clinical target volume
DFS: Disease-free survival
ECOG: Eastern Cooperative Oncology Group
GTV: Gross tumor volume
IMRT: Intensity modulated radiation therapy
LAHNC: Locally advanced head and neck cancer
OS: Overall survival
PTV: Planning target volume
SeqB: Sequential boost
SIB: Simultaneous integrated boost
VMAT: Volumetric-modulated arc therapy
