# Supplementary material for: A comparative analysis between sequential boost and integrated boost intensity-modulated radiation therapy with concurrent chemotherapy for locally-advanced head and neck cancer
Source: Radiat Oncol. 2017 Jan 13;12:13. doi: 10.1186/s13014-016-0756-x (PMC5237132; doi:10.1186/s13014-016-0756-x)
Supplement: Additional file 1: Table S1. — Chemotherapies Utilized in Sequential Boost Cohort. Table S2. Acute toxicity in sequential and integrated boost cohorts among patients who received 5 or more cycles of concurrent chemotherapy. (DOCX 12 kb) [file 13014_2016_756_MOESM1_ESM.docx]

Additional file 1

Table S1. Chemotherapies Utilized in Sequential Boost Cohort

| **Induction** | **Number (%)** |
| --- | --- |
| None  Carboplatin/Paclitaxel  Carboplatin/Paclitaxel/Cetuximab  Docetaxel/Cisplatin/5-FU (TPF)  Cisplatin/5-FU  Oxaliplatin/Alimta | 15 (22)  35 (51)  6 (9)  9 (13)  1 (1)  2 (3) |
| **Concurrent** | **Number (%)** |
| Carboplatin/Paclitaxel  Carboplatin  Cisplatin  Variable | 64 (95)  1 (1)  1 (1)  2 (3) |

Table S2. Acute toxicity in sequential and integrated boost cohorts among patients who received 5 or more cycles of concurrent chemotherapy.

| **Sequential Boost**  Dermatitis (n=47)  Mucositis (n=47)  Xerostomia (n=44)  Dysphagia (n=48) | Grade 0 | Grade 1 | Grade 2 | Grade 3 | Grade 4 |
| --- | --- | --- | --- | --- | --- |
|  | 0%  4%  11%  2% | 0%  9%  50%  4% | 34%  19%  36%  40% | 64%  66%  2%  54% | 2%  2%  0%  0% |
| **Integrated Boost**  Dermatitis (n=74)  Mucositis (n=70)  Xerostomia (n=67)  Dysphagia (n=73) | Grade 0 | Grade 1 | Grade 2 | Grade 3 | Grade 4 |
|  | 0%  1%  0%  0% | 5%  3%  55%  1% | 16%  11%  45%  18% | 72%  83%  0%  78% | 7%  1%  0%  3% |
